# ΔNp63α-mediated epigenetic regulation in keratinocyte senescence

**DOI:** 10.1080/15592294.2023.2173931

**Published:** 2023-02-09

**Authors:** Linghan Kuang, Chenghua Li

**Affiliations:** a Department of Laboratory Medicine, West China Second University Hospital, Sichuan University, Chengdu 610041, China; b Key Laboratory of Birth Defects and Related Diseases of Women and Children (Sichuan University), Ministry of Education, Chengdu 610041, China; c Center of Growth, Metabolism and Aging, Key Laboratory of Biological Resources and Ecological Environment of Ministry of Education, College of Life Sciences, Sichuan University, Chengdu 610065, China

**Keywords:** Keratinocyte, skin aging, cellular senescence, cell proliferation, ΔNp63α, epigenetic regulation, chromatin remodeling

## Abstract

Keratinocyte senescence contributes to skin ageing and epidermal dysfunction. According to the existing knowledge, the transcription factor ΔNp63α plays pivotal roles in differentiation and proliferation of keratinocytes. It is traditionally accepted that ΔNp63α exerts its functions via binding to promoter regions to activate or repress gene transcription. However, accumulating evidence demonstrates that ΔNp63α can bind to elements away from promoter regions of its target genes, mediating epigenetic regulation. On the other hand, several epigenetic alterations, including DNA methylation, histone modification and variation, chromatin remodelling, as well as enhancer-promoter looping, are found to be related to cell senescence. To systematically elucidate how ΔNp63α affects keratinocyte senescence via epigenetic regulation, we comprehensively compiled the literatures on the roles of ΔNp63α in keratinocyte senescence, epigenetics in cellular senescence, and the relation between ΔNp63α-mediated epigenetic regulation and keratinocyte senescence. Based on the published data, we conclude that ΔNp63α mediates epigenetic regulation via multiple mechanisms: recruiting epigenetic enzymes to modify DNA or histones, coordinating chromatin remodelling complexes (CRCs) or regulating their expression, and mediating enhancer-promoter looping. Consequently, the expression of genes related to cell cycle is modulated, and proliferation of keratinocytes and renewal of stem cells are maintained, by ΔNp63α. During skin inflammaging, the decline of ΔNp63α may lead to epigenetic dysregulation, resultantly deteriorating keratinocyte senescence.

## Introduction

The largest organ of the human body, skin, acts as a protective barrier on our surface. It is also responsible for maintaining homoeostasis of various substances as well as sensory perception and temperature regulation [1,2]. Keratinocyte is the predominant cell type in the epidermis, which is the outermost layer of the skin. Senescence of keratinocytes is an important part of skin ageing, which may impair the functions of skin. Numerous studies show that epigenetic alterations are involved in skin ageing and cell senescence [3–5]. p53 family proteins are key transcription factors controlling cell cycle [6]. p63 belongs to p53 gene family and encodes multiple protein isoforms, among which ΔNp63α is the predominant species expressed in the skin tissue [7]. As a key transcription factor in keratinocyte differentiation and renewal, ΔNp63α plays key roles to maintain proliferation of keratinocytes and to prevent them from undergoing senescence. It is traditionally accepted that ΔNp63α exerts proliferative functions via binding to promoter regions of various cell cycle arresting genes (e.g., Pten, p16^INK4a^ and p21^WAF1/CIP1^) and consequently downregulating their expression [7–9]. Recently, ΔNp63α has been found to act as a pioneer transcription factor to regulate gene expression via binding to regions other than promoters [10,11]. In this review, we focus on the epigenetic regulation of ΔNp63α in preventing keratinocyte senescence.

## ΔNp63α and senescence of keratinocytes

The skin consists of three layers, the epidermis, dermis, and hypodermis, each of which is separate but functionally interdependent. The outermost layer, epidermis, is predominantly composed of keratinocytes, which provide hardness and moisture-lock properties to the skin [1]. Keratinocytes are continuously proliferating to renew the outer skin barrier. They migrate upwards and differentiate into cells that comprise the epidermis [12–14].

**Figure 1.**
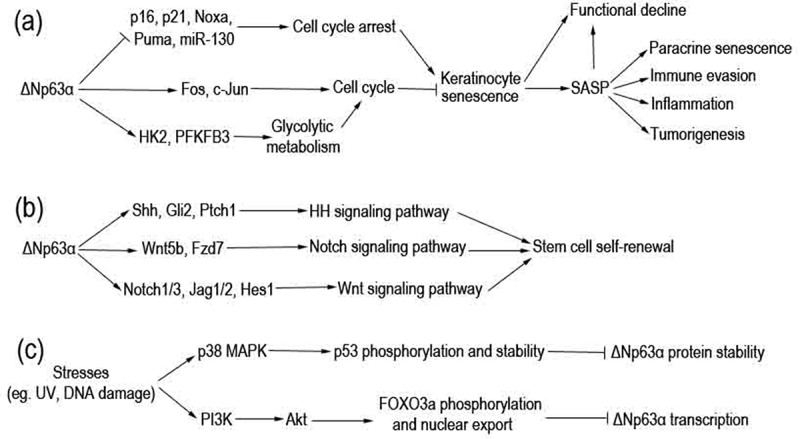
Pathways through which ΔNp63α regulates keratinocyte senescence and ΔNp63α is regulated. (a) ΔNp63α regulates transcription of diverse genes involved in cell cycle. Consequently, keratinocyte senescence and its detrimental outcomes are prevented. (b) ΔNp63α transactivates multiple genes to activate Hh, Notch, and Wnt signaling pathways. In turn, stem cell self-renewal is maintained. (c) Various stresses can stimulate p38 MAPK or PI3K/Akt pathways, resulting in downregulation of ΔNp63α at the mRNA or protein level.

Skin undergoes intrinsic or chronological ageing, as a consequence of internal and external changes due to passage of time [15]. Owing to extensive contact with the outside environment, epidermal tissues (especially keratinocytes) are continuously exposed to diverse external stimuli, which can cause inflammaging [16–18]. Alongside inflammaging of the skin, the chronic low-level pro-inflammatory cytokines, in concert with the external stimuli, may induce and deteriorate senescence of keratinocytes and other skin-resident stromal cells. As a continuously renewing epithelium, the epidermis relies heavily on the proliferative potential of keratinocytes to work as a functional barrier of our body [19]. Senescence of keratinocytes may lead to decline of their physiological functionality, which accelerates the ageing process of skin tissues [13,20]. In particular, these senescent keratinocytes produce a complex secretome, which is termed senescence-associated secretory phenotype (SASP) and leads to some detrimental effects, such as paracrine senescence, immune evasion, inflammation, and tumorigenesis (depicted as Figure 1a) [21].

**Figure 2.**
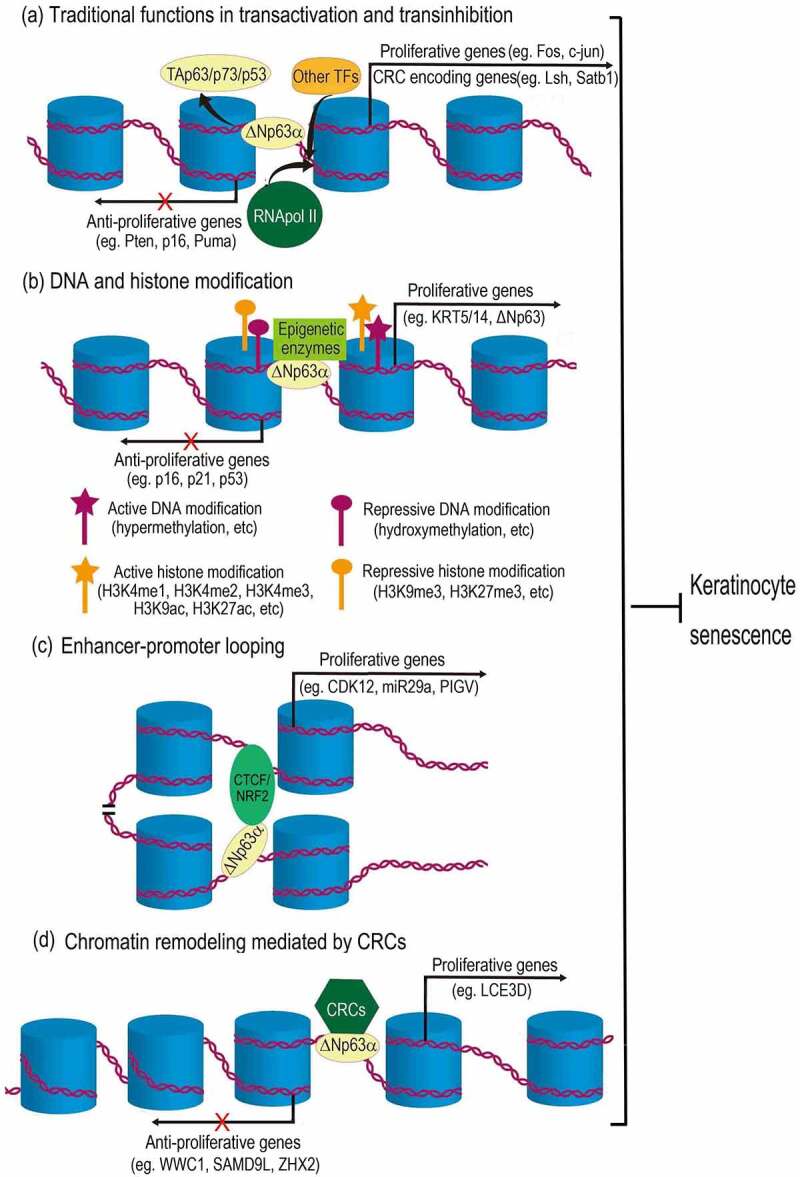
Mechanisms of ΔNp63α-mediated epigenetic regulation in preventing keratinocyte senescence. (a) As a traditional transcription factor, ΔNp63α binds to promoter regions, antagonizing its homologues (TAp63, p73 or p53), as well as recruiting other transcription factors (TFs) and RNA polymerase II (RNApol II), regulating genes related to cell cycle and chromatin remodeling complex (CRC). (b) ΔNp63α recruits diverse epigenetic enzymes (e.g., DNMT3A, KMT2D, HDAC1/2), leading to either active or repressive modification of DNA and histones. (c) ΔNp63α cooperates with CTCF or NRF2 to mediate enhancer-promoter looping. (d) ΔNp63α can also recruit CRCs (e.g., BAF, ACTL6A, SRCAP) to change chromatin configurations, which may either increase or decrease DNA accessibility for transcription machinery. Resultantly, proliferative genes are upregulated, while anti-proliferative genes are downregulated. Therefore, proliferation of keratinocytes is maintained and senescence is prevented.

As an indispensable transcription factor in keratinocytes, ΔNp63α plays a master role in maintaining proliferation and preventing senescence of these cells [19]. ΔNp63α is encoded by p63 gene, which belongs to the p53 transcription factor gene family and produces multiple protein isoforms [7,22]. Among them, ΔNp63α is the predominant isoform in somatic cells, especially in keratinocytes [23–25]. As depicted in Figure 2a, ΔNp63α directly binds to p63-responsive elements (p63REs) in promoter regions of its target genes to mediate either transcriptional activation or inhibition in different scenarios [7,26]. These p63REs can also be recognized by other p53 family members, including p53, TAp63 and p73 proteins. Competitive association as well as heteromerization of ΔNp63α can also downregulate these target genes of p53 family transcription factors [7,8,19]. Data from our group and other labs demonstrate that ΔNp63α downregulates a batch of cell-cycle arrest-related genes including p21^WAF1/CIP1^, p16^INK4a^, miR-130, MM1, Noxa and Puma, maintaining cell proliferation and survival (depicted as Figure 1a) [27–32]. On the other hand, there is also evidence that ΔNp63α maintains keratinocyte proliferative capacity via upregulating cell cycle genes such as Fos and c-Jun [33,34], as well as genes involved in glycolytic metabolism, such as hexokinase 2 (HK2) and 6-phosphofructo-2-kinase/fructose-2,6-bisphosphatase 3 (PFKFB3) (depicted as Figure 1a) [35,36].

Epidermal stem cells are important for replacing the cells lost during keratinocyte differentiation and senescence. It is documented that ΔNp63α keeps the regenerative capacity of stratified epithelial structure via maintaining epithelial stemness [37]. ΔNp63α is essential for the asymmetric division of epithelial stem cells to maintain the stem cell pool [38], which can be exhausted via symmetric division into transit-amplifying cells in the absence of p63 gene, resulting in epithelial defects [39–41]. ΔNp63α may contribute to the stemness phenotype via controlling multiple signalling pathways (depicted as Figure 1b): (1) transactivating Shh, Gli2 and Ptch1 to activate the Hedgehog (Hh) pathway [42,43]; (2) stimulating the Notch pathway via positively regulating the expression of a number of components of this pathway, including Notch1, Notch3, Jag1, Jag2, and Hes1 [39,44–47]; (3) activating Wnt signalling pathway through the direct upregulation of transcription of the Fzd7 receptor as well as the Wnt5a ligand [48]. Via modulating these signalling pathways in different scenarios, ΔNp63α governs the balance between self-renewing and transit-amplifying cells [37,49].

The essential roles of ΔNp63α in antagonizing keratinocyte senescence are supported by data from mouse models and clinical studies. Keyes WM et al. found that accelerated skin ageing is induced in mice conditionally deleted with p63 in the epidermis, where ΔNp63α is the predominant isoform [50]. Melino G group demonstrated that the expression of p63 in skin keratinocytes of elderly people is significantly lower than that of youths, and downregulation of ΔNp63α accelerates skin ageing [19]. In one of our recent studies, we found that ΔNp63α is downregulated in either mouse skin or human keratinocytes during photoaging induced by ultraviolet B (UVB) [51].

Downregulation of ΔNp63α during skin ageing may be due to the activation of Akt/Foxo3a, p38 MAPK or other signalling pathways mediated by ROS and DNA damage, according to the experimental results of our laboratory and other research groups (depicted as Figure 1c) [31,51–53]. Ultraviolet radiation (UVR) can activate p38 MAPK pathway, which in turn phosphates and stabilizes p53 in keratinocytes; consequently, p53 binds to ΔNp63α and direct a caspase-1-mediated degradation of the latter protein [52]. In our previous studies, we demonstrated that the treatment with DNA damage drugs down-regulates ΔNp63α at the mRNA level, which is independent of p53 [31]. Recently, it is reported that UVB radiation can activate PI3K/Akt pathway [54], which may increase FOXO3a phosphorylation and nuclear export, resulting in downregulation of ΔNp63α transcription [53].

## Epigenetics in cell senescence

Epigenetics is a class of mechanisms generating heritable phenotypic changes independently of DNA sequence changes. Mechanisms of epigenetics include DNA and histone modifications, chromatin remodelling, as well as other processes such as enhancer-promoter looping. Via these mechanisms, environment can regulate gene expression to produce visible phenotypes [55,56]. As a state of cell cycle arrest is regulated by a network of molecules, cell senescence is driven by diverse epigenetic changes [5,57].

### DNA methylation

The templates of transcription, DNA, randomly contains cytosines, which can be potentially methylated by DNA methyltransferases (DNMTs), i.e., DNMT1, DNMT3A and DNMT3B. 5-methyl cytosine (5mC) is enriched in CpG dinucleotides and directly affects the ability of transcription factors and other DNA binding proteins to access DNA. Therefore, hypermethylation of CpGs in gene promoters generally results in silencing or low expression of downstream genes, while hydroxymethylation upregulates gene transcription [58,59].

It has been reported that senescence-associated DNA methylation alterations accumulate during cell senescence and consequently cause cell cycle arrest [57]. Mislocalization or repression of DNMT1 contributes to senescence-associated DNA hypomethylation [60,61]. On the other hand, senescence-associated heterochromatin foci (SAHF) may recruit DNA methyltransferases (DNMTs) to focal sites to induce senescence-associated focal hypermethylation [62,63]. These DNA methylation alterations promote replicative senescence via multiple mechanisms [64], including upregulating p16^INK4a^ and p21^WAF1/CIP1^ [65], downregulating telomerase reverse transcriptase (TERT) expression and telomerase activity [66], as well as recruiting polycomb repressive complex 2 (PRC2) [67].

### Histone modification

In eukaryotic chromatin, DNA strands are packaged into nucleosomes by histones including H2A, H2B, H3, and H4 [68]. Diverse modifications, including different types of acetylation and methylation at specific residues of histones, can change chromatin figuration and regulate gene expression via affecting DNA accessibility for the transcription machinery [69].

It is well documented that histone-associated epigenetic processes occur during senescence. These processes include modification, variation and depletion of histones [57]. It was reported that telomere shortening triggers histone depletion and gives rise to an open chromatin configuration, consequently enhancing RNA polymerase II elongation rates as well as resulting in pre-mRNA splicing defects in senescence [70,71]. In replicative senescence and some types of stress-induced premature senescence (SIPS), histone modification changes have been detected, which include global decreases in H3 lysine 4 trimethylation (H3K4me3), H3 lysine 9 trimethylation (H3K9me3), H3 lysine 27 trimethylation (H3K27me3) and H4 lysine 16 acetylation (H4K16Ac), as well as comprehensive increases in H3 lysine 9 acetylation (H3K9Ac) and H4 lysine 20 trimethylation (H4K20me3) [72–74]. These alterations mediated by specific enzymes can affect its affinity to DNA strains and condensation state of nucleosomes, eventually modulating the expression and effects of cell cycle-related proteins including p14^ARF^ [75,76], p15^INK4b^ [75,76], p16^INK4a^ [75–77], p21^WAF1/CIP1^ [78,79], and Rb [77,80], as well as inflammaging-related cytokines such as IL-6 and IL-8 [78,79]. In turn, they lead to telomere shortening or dysfunction [81,82], proliferation suppression [83,84], and senescence-associated secretory phenotype (SASP) [78,79,85].

### Structural transformation of chromatin mediated by chromatin remodeling complexes

Chromatin remodelling complexes (CRCs), also named as remodellers, are needed to fully package the genome, to specialize chromatin regions, and to provide regulated DNA accessibility in packaged regions. Four classes of CRCs are found in eukaryotes [86,87]: switching defective/sucrose non-fermenting (SWI/SNF) family; imitation switch (ISWI) family; chromodomain, helicase, DNA binding (CHD) family; inositol requiring 80 (INO80) family. CRCs utilize ATP hydrolysis to alter histone-DNA contacts and modulate sliding of histone octamers across DNA, association between them, as well as the position of nucleosomes [88,89]. In replicative senescence, these CRC-mediated structural transformations of chromatin can induce cell cycle arrest via upregulating expression of p16^INK4a^, p21^WAF1/CIP1^, and p53 [90–93].

### Enhancer-promoter looping

Chromatin structure in mammals can also regulate transcription by modulating three-dimensional interactions between enhancers and promoters, which can be facilitated by chromatin loops [94]. CCCTC-binding factor (CTCF) is a transcription factor which works together with the cohesion complex to drive the formation of chromatin loops [95]. Recently, Olan I et al reported that enhancer-promoter interactions are extensively altered during cell senescence. These rewired enhancer-promoter interactions may modulate the expression of genes related to cell senescence, such as IL1B [96].

## ΔNp63α-mediated epigenetic regulation and keratinocyte senescence

As a traditional transcription factor, ΔNp63α is well established to bind to canonical p53 DNA binding sites in promoter regions of its target genes and thus compete with TAp63/p73/p53, resulting in transinhibition (Figure 2a). ΔNp63α-promoter association can also activate gene expression via recruiting other transcription factors (TFs) and RNA polymerase II (RNApol II) (Figure 2a) [7]. Recently, accumulating data demonstrate that ΔNp63α directly binds to DNA regions other than promoters and consequently regulates expression of genes related to keratinocyte differentiation and proliferation [10,97–99]. This indicates that, like its homologue p53, ΔNp63α may also function as a pioneer transcription factor to modulate chromatin structure and to regulate diverse biological processes, including keratinocyte senescence [8,11,100].

## ΔNp63α regulates DNA modification to promote keratinocyte proliferation

Using epigenome profiling of differentiating human primary epidermal keratinocytes, Kouwenhoven EN et al. found that ΔNp63α binds to a batch of enhancers [10]. Rinaldi L et al found that via binding to the enhancers, ΔNp63α recruits DNA methyltransferase 3A (DNMT3A) to the centre of the enhancers. Then, TET2 mediates the sequential hydroxymethylation of cytosine there. This can activate the expression of diverse genes, such as KRT5, KRT14 and ΔNp63 itself. These genes are related to differentiation and proliferation of epidermal cells [58,101]. These data indicate that ΔNp63α may promote keratinocyte proliferation and antagonize senescence via facilitating cytosine hydroxymethylation at the enhancers of proliferative genes and upregulating their expression (depicted as Figure 2b). It remains to be further investigated whether ΔNp63α regulates other types of DNA modification via recruiting other enzymes.

## ΔNp63α regulates histone modifications to prevent keratinocyte senescence

Recent investigations suggest that ΔNp63α affects histone acetylation and nucleosome repositioning and changes DNA accessibility to regulate gene expression (depicted as Figure 2b). Among the ΔNp63α-bound enhancers identified by Kouwenhoven EN et al, a half is active as defined by histone modification H3K27ac, which is an active enhancer mark. These data indicate that ΔNp63α upregulates nearby genes (e.g., KRT5, KRT10 and TGM1) related to keratinocyte proliferation and differentiation via promoting histone acetylation [10]. In another study, Hamdan FH et al identified numerous ΔNp63α-occupied distal elements away from the transcription start sites of genes in pancreatic cancer cells. Many of these elements are active enhancers intersected with H3K27ac mark and locate in open chromatin regions identified by assay for transposase-accessible chromatin sequencing (ATAC-seq). This distal pattern of occupancy implies the function of ΔNp63α in enhancer activation to promote cell proliferation [102]. In line with both reports, Yu X et al found that ΔNp63α can bind to inaccessible chromatin regions, which is characterized by unmodified histones; after ΔNp63α binding, histone modifications occur in these regions [11]. These data indicate that ΔNp63α induces proliferative genes via increasing chromatin accessibility and this effect may be exerted in various situations.

ΔNp63α-induced histone modifications may be not limited to increasing chromatin accessibility. Data from Yu X et al show that histone modifications induced by ΔNp63α include not only transcriptional active histone modifications (H3K9ac, H3K27ac, H3K4me1, H3K4me2, H3K4me3), but also repressive ones (H3K27me3 and H3K9me3) [11]. Consistent with the repression effect of p63 binding, Pattison JM et al found that p63 induces trimethylation of histone H3 lysine 27 (H3K27me3) and closes chromatin accessibility in keratinocytes [98].

These modifications may be due to ΔNp63α-mediated recruitment of epigenetic enzymes. Lin-Shiao E et al found that the histone methyltransferase KMT2D interacts with ΔNp63α on chromatin and at a broad array of enhancers, resulting in histone modifications, including H3 lysine 4 monomethylation (H3K4me1) and H3 lysine 27 acetylation (H3K27ac), in keratinocytes. This may maintain transcription of hundreds of genes (e.g., KRT5, KLF4 and ZNF750), which are essential for p63 to keep proliferative capacity of keratinocytes through positively regulating genes critical for epithelial development, differentiation, and adherence [97]. Ramsey MR et al reported that ΔNp63α recruits histone deacetylases HDAC1 and HDAC2 to erase the acetylation of histones. This histone deacetylation increases histone-DNA affinity and mediates transcriptional repression of genes (e.g., Puma) responsible for cell proliferation and tumorigenesis [103,104].

### ΔNp63α coordinates with chromatin remodeling complexes or regulates their expression to prevent keratinocyte senescence

ΔNp63α can also coordinate chromatin remodelling complexes (CRCs) to orchestrate the remodelling (depicted as Figure 2d). BRG1/BRM-associated factor (BAF) belongs to the SWI/SNF CRC family [105]. Using assay for transposase-accessible chromatin with high throughput sequencing (ATAC-seq), Bao X et al revealed that BAF and p63 mutually recruit each other to maintain 14,853 open chromatin regions and cooperatively position nucleosomes, resultantly recruiting transcriptional machinery and controlling transcription during epidermal differentiation. Chromatin remodelling coordinately mediated by p63 and BAF upregulates multiple genes related to epidermal differentiation and cell proliferation, including LCE3D, KRT1 and KRT10 [106].

The interaction and cooperation between ΔNp63α and CRCs in chromatin remodelling is also found in tumorigenesis. ΔNp63α may interact with actin-like protein 6A (ACTL6A), which is a subunit of SWI/SNF CRC and co-amplified with ΔNp63α in head and neck squamous cell carcinoma (HNSCC); this collaboration leads to a downregulation of tumour suppressors such as WWC1 and GPRC5A [107]. In addition, ΔNp63α can participate in the formation of another CRC called SRCAP (SNF2-related CBP Activator Protein), which belongs to the INO80 family and facilitates the substitution of histone H2A to its variant H2A.Z in nucleosomes; as a result, anti-proliferative genes (e.g., SAMD9L, ZHX2, and IGFBP3) are repressed at transcription level [108–110]. It remains obscure whether ΔNp63α modulates keratinocyte senescence via these mechanisms.

Apart from the direct interactions with CRCs, ΔNp63α can also modulate chromatin remodelling via regulating gene expression of multiple remodellers or related proteins (depicted as Figure 2a&d). Keyes WM et al previously reported that ΔNp63α drives proliferation of skin stem cells via transactivating lymphoid-specific helicase (LSH) [111], which belongs to the SNF2 family of chromatin-remodelling ATPase and promotes cell proliferation in multiple scenarios [112,113]. Intriguingly, Mardaryev AN et al found that ΔNp63α can bind to intron 1 of BRG1 gene and upregulate its transcription in epidermal progenitor cell during skin development [114]. As mentioned above, BRG1 is the core subunit of SWI/SNF-like BAF remodeller, which directly binds to p63 protein and cooperatively modulates DNA accessibility during epidermal differentiation [106]. These reports indicate that ΔNp63α may promote BAF-mediated chromatin remodelling from two dimensions: transactivating BRG1 gene and coordinating with the BAF complex. In addition, Fessing MY et al found that ΔNp63α controls expression of genes encoding several regulators of the higher-order chromatin structure and ATP-dependent chromatin remodelling [115]. Among which, special AT-rich binding protein 1 (SATB1) acts as a docking site for several chromatin remodelling enzymes to regulate gene expression [116]. According to Fessing MY’s data, ΔNp63α directly binds to the promoter of SATB1 gene and upregulates its expression in keratinocytes, hence controlling chromatin remodelling during development of the epidermis [115].

## ΔNp63α prevents keratinocyte senescence via mediating enhancer-promoter looping

Several recent studies suggest that ΔNp63α promotes keratinocyte proliferation via mediating enhancer-promoter looping in cooperation with other transcription factors (depicted as Figure 2c). Qu J et al found that ΔNp63α and CTCF are cooperatively involved in chromatin looping to regulate epidermal genes [117]. CTCF is found to consistently bind to promoters of epidermal genes (e.g., PIGV and KRT5) [118]. This consistent association of CTCF acts as a barrier for gene expression under most scenarios [94]. However, the p63-CTCF cooperation assists the looping of p63-bound enhancers to gene promoters and turns them on during keratinocyte differentiation and proliferation [117]. It is reported that CTCF mediates chromatin remodelling via physically interacting with BRD9, which is a component of non-canonical BAF complex [119]. It remains unclear whether this SWI/SNF chromatin remodelling complex is involved in p63-CTCF cooperation in enhancer-promoter looping. According to a recent study conducted by Kurinna S et al, p63 can also mediate enhancer-promoter looping in cooperation with another transcription factor, nuclear factor erythroid 2-related factor 2 (NRF2). This can activate genes including CDK12 and miR29a and promotes keratinocyte proliferation in the epidermis [99].

## Discussion

Keratinocytes compose the epidermis and keep exuberant proliferating capacity to renew this skin layer [19]. Senescence of keratinocytes heavily impairs skin functionality and seriously deteriorates the skin ageing process [13,20]. As a crucial transcription factor in epidermal differentiation and cell proliferation, ΔNp63α has been reported to play key roles in delaying keratinocyte senescence [19]. Generally, ΔNp63α is assumed to specifically bind to elements in the promoter regions of its target genes to activate (e.g., Fos, c-Jun, HK2, and KRT14) or repress (e.g., Pten, p16^INK4a^, p21^WAF1/CIP1^ and Puma) their expression (depicted as Figure 2a) [7,9]. As a consequence, ΔNp63α promotes epidermal differentiation and keratinocyte proliferation.

Since accumulating data demonstrate that ΔNp63α binds to regions away from promoters (e.g., enhancers and introns) [10,11,120], it may mediate epigenetic regulation to prevent keratinocyte senescence via multiple mechanisms (depicted as Figure 2a~d): (1) ΔNp63α recruits epigenetic enzymes (e.g., DNMT3A, KMT2D and HDAC1/2) to modify DNA or histones, resulting in either increasing or decreasing DNA accessibility for transcription machinery (Figure 2b) [10,58,102]; (2) ΔNp63α can also mediate enhancer-promoter looping in cooperation with other transcription factors (TFs), such as CTCF and NRF2 (Figure 2c) [99,117]; (3) ΔNp63α physically interacts with and coordinates chromatin remodelling complexes (e.g., BAF, ACTL6A and SRCAP; depicted as Figure 2d) [106–108,111,114,115]; (4) ΔNp63α transactivates genes related to chromatin remodelling and indirectly promotes the remodelling (Figure 2a and 2d). Via these mechanisms, ΔNp63α downregulates anti-proliferative genes (e.g., p16^INK4a^, p21^WAF1/CIP1^, Puma, p53, WWC1, SAMD9L, and ZHX2), as well as upregulates proliferative genes (e.g., Fos, c-Jun, HK2, PFKFB3, KRT5, KRT14, ΔNp63, CDK12 and miR29a). Hence, ΔNp63α promotes proliferation of keratinocytes and prevents them from senescence (depicted in Figure 2). It is worth noting that some genes (e.g., KRT5, KRT14, p16^INK4a^ and p21^WAF1/CIP1^) are commonly regulated by the abovementioned mechanisms, indicating that these ΔNp63α-mediated epigenetic regulation mechanisms may be intertwined. During skin inflammaging, the decline of ΔNp63α induced by various factors may lead to dysregulation of chromatin remodelling, consequently deteriorating senescence of keratinocytes. It is worthful to study how these epigenetic changes differentially regulate proliferative or anti-proliferative genes. The genes regulated by ΔNp63α during keratinocyte senescence are also remained to be systematically investigated.

It has been reported that some chemicals (e.g., resveratrol, aspirin and metformin) exert anti-senescence effects in multiple cell types via regulating epigenetic alterations [5,57,121–125]. More to the point, epigenetic drugs (e.g., DNMT inhibitor RG108 and histone methyltransferase Smyd3 inhibitor EPZ031686) can mitigate senescence phenotype in vitro and in vivo [60,126]. Given the roles of ΔNp63α-mediated chromatin remodelling against keratinocyte senescence, it is possible to delay skin ageing via therapeutic interventions with epigenetic drugs.

## Data Availability

Not applicable.

## Article highlights

- Knowledges of ΔNp63α and keratinocyte senescence were introduced
- General epigenetic alterations in cellular senescence were summarized
- Mechanisms of ΔNp63α-mediated epigenetic regulation in preventing keratinocyte senescence, including DNA and histone modification, enhancer-promoter looping, directly and indirectly promoting chromatin remodelling, were reviewed.
